# Influence of grain boundary characteristics on thermal stability in nanotwinned copper

**DOI:** 10.1038/srep31410

**Published:** 2016-08-12

**Authors:** Rongmei Niu, Ke Han, Yi-feng Su, Tiglet Besara, Theo M. Siegrist, Xiaowei Zuo

**Affiliations:** 1 National High Magnetic Field Laboratory, Florida State University, 1800 E. Paul Dirac Drive, Tallahassee, FL32310, USA; 2 Department of Chemical and Biomedical Engineering, Florida State University, 32310, USA; 3 Key Laboratory of Electromagnetic Processing of Materials (Ministry of Education), Northeastern University, Shenyang 110004, China

## Abstract

High density grain boundaries provide high strength, but may introduce undesirable features, such as high Fermi levels and instability. We investigated the kinetics of recovery and recrystallization of Cu that was manufactured to include both nanotwins (NT) and high-angle columnar boundaries. We used the isothermal Johnson-Mehl-Avrami-Kolmogorov (JMAK) model to estimate activation energy values for recovery and recrystallization and compared those to values derived using the non-isothermal Kissinger equation. The JMAK model hinges on an exponent that expresses the growth mechanism of a material. The exponent for this Cu was close to 0.5, indicating low-dimensional microstructure evolution, which is associated with anisotropic twin coarsening, heterogeneous recrystallization, and high stability. Since this Cu was of high purity, there was a negligible impurity-drag-effect on boundaries. The twin coarsening and heterogeneous recrystallization resulted from migration of high-angle columnar boundaries with their triple junctions in one direction, assisted by the presence of high concentration vacancies at boundaries. Analyses performed by electron energy loss spectroscopy of atomic columns at twin boundaries (TBs) and in the interior showed similar plasma peak shapes and L3 edge positions. This implies that values for conductivity and Fermi level are equal for atoms at TBs and in the interior.

Nanotwinned (NT) materials have been the subject of intensive study for years because of their unique microstructure and properties^1^,^2^,^3^,^4^. Engineered coherent and stable internal boundaries allow for substantial strength and optimum electrical conductivity while preserving acceptable levels of ductility^2^. All of these desirable properties stem from the nature of high densities of {111} 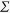
3 twin orientation in fcc metals. Atomic planes at a coherent twin boundary (CTB) fit perfectly into both jointed grains, have low energy, and are thus immobile at room temperatures and below. By contrast, atomic planes at the vicinity of incoherent twin boundary (ITB) become distorted. ITB energy is therefore higher and ITB is more mobile than CTB. Our previous studies have demonstrated that, at room temperature, as-deposited NT Cu (i.e., with CTBs) remains stable over years, while 15%-strained NT Cu becomes unstable within a month^5^,^6^,^7^,^8^. This instability is a result of ITB-induced recovery and recrystallization that in turn leads to the softening^5^.

The as-deposited NT Cu has columnar structure, with the columns running along the growth direction, perpendicular to the film interface. Each column consists of high density CTBs. When column size is small, a pronounced feature is the presence of columnar boundaries (CLBs) and a high density of triple junctions (TJs, where TBs meet CLBs) due to nano-scale twin spacing. Previous TEM *in-situ* experiments on thin film Cu have demonstrated emission of dislocations from TJs^9^. In nanocrystalline materials, grain boundary TJs have been found to exert energetic or accelerating effect on grain growth^10^. For polycrystals with a grain size about 50 nm, the driving force from the TJs is comparable with that from grain boundaries themselves^10^. These observations can be used to explain later research showing that after 800 °C annealing for 1 h, the columnar grain size in NT Cu rapidly rises from 50 nm to 500 nm, and twin lamellar thickness increases from 4 nm to 20 nm^4^. We speculate that TJs, which connect twin boundaries and columnar boundaries, play a role in columnar grain size growth in all NT materials.

Most of the CLBs in NT Cu are random high-angle boundaries (HABs)^7^,^8^,^11^. Recent studies^12^,^13^,^14^,^15^ on the influence of orientation differences on mobility confirm that the mobility of HABs is about 100 to 1,000 times greater than that of low-angle boundaries. HABs are usually incoherent, thus less obstacle to sliding when compared to low-energy CTBs. Previous investigations have demonstrated that the presence of large fractions of high-angle boundaries enhances nucleation of new crystals^14^,^16^, thus benefitting recovery and recrystallization^7^ and reducing thermal stability.

For the commercial application of NT metals, stability is a main concern for their applications. We speculate that a high density of HABs and TJs would disturb the thermal stability of any CTBs in the vicinity. Few investigations have been conducted on the kinetics of recovery and recrystallization in NT Cu, though some work has been carried out on the HAG Cu^17^,^18^,^19^,^20^,^21^,^22^. This paper reports our studies of the kinetics of recovery and recrystallization of NT Cu. We analyzed the activation energy in isothermal and non-isothermal models using both hardness evaluation and differential scanning calorimetry (DSC). We investigated CLBs (especially HABs) with respect to their microstructural evolution after annealing at various time and temperatures. We probe the atomic structure at the twin boundaries using atomic resolution Z-contrast imaging generated by high angle annual dark field (HAADF) scanning transmission electron microscopy (STEM). Based on these studies, we proposed an approach to controlling and optimizing microstructural stability in NT materials.

## Results

### Hardness evolution and thermal analyses for isothermal annealing

**Figure 1:**
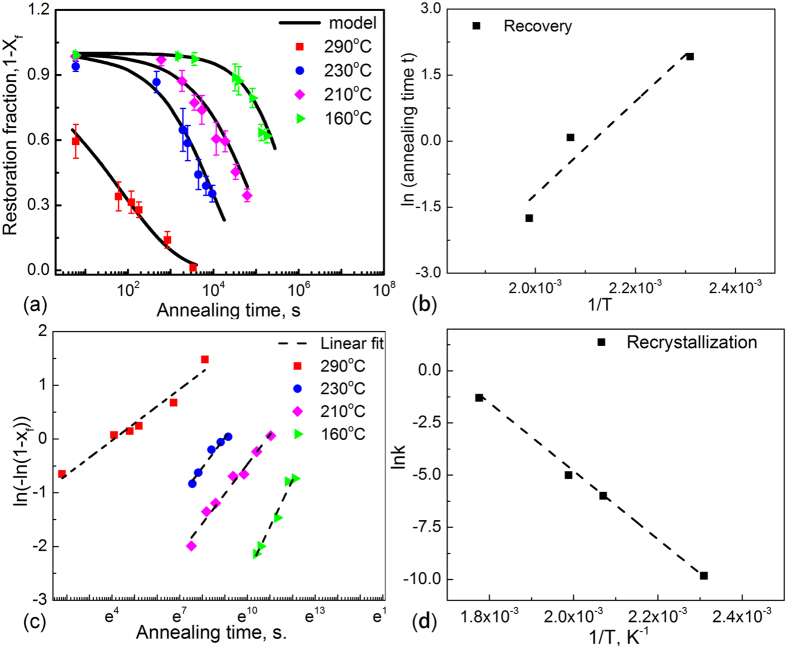
Plots showing isothermal kinetics of recovery and recrystallization in as-deposited NT Cu at different temperatures. (**a**) Restoration fraction as a function of annealing time (seconds or s) for different temperatures in Celsius (°C). The different symbols are experimental data. Systematic errors are indicated by error bars. The solid black lines show a fit (Eq. 6) of the *1−X*_*f*_ vs *t* data using parameters calculated (*k*_*0i*_*, n*, and *E*) in Table 2. The average error of time *t* is ±8 s. (**b**) Annealing time (*lnt*) vs the reciprocal temperatures for *X*_*f*_ = 0.9%. The activation energy E_ry_ is calculated by the linear regression, see details in Supplementary S1. (**c**) Logarithmic plot of *ln (−ln(1−X*_*f*_)) vs. annealing time *t* during recrystallization. (**d**) Recrystallization activation energy plot of *ln k* versus the reciprocal temperatures, the unit of *k* is *s*^*−n*^.

Both recovery and recrystallization can be measured by changes in yield stress, resistivity or hardness^23^,^24^,^25^. Hardness decreases have been discussed in numerous studies performed in our lab and others. In our past studies, the focus has been on as-deformed NT Cu^5^, but in this present study, we turn our attention instead to as-deposited NT Cu. Recovery and recrystallization in NT Cu is described as follows^13^:


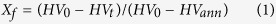


where *X*_*f*_ is the transformed volume fraction after a time *t*, *HV*_0_ is the hardness of as-deposited NT Cu, *HV*_*t*_ is the hardness of annealed NT Cu at time *t*, and *HV*_*ann*_ is the hardness of fully recrystallized NT Cu. Figure 1(a) depicted the kinetic evolution of hardness in as-deposited NT Cu obtained after isothermal recrystallization treatments carried out at 160 °C, 210 °C, 230 °C, and 290 °C. The curves display a shallow decrease in the early stage of annealing and a steep decrease in the later stages. The higher the temperature, the shorter the early stage and the longer the later stage.

For the kinetics of recovery, a relationship based on microstructure has been developed^26^:


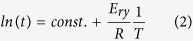


where *t* represents the time needed to reach the softened fraction *X*_*f*_, *E*_*ry*_ is the activation energy for recovery (kJ/mol), *R* is the gas constant (8.31 J/mol·K), and *T* is the absolute temperature (K). For a given softened fraction at various temperatures, the required time *t* can be determined from the curve of *X*_*f*_ versus t (Fig. 1 (a)). The activation energy (*E*_*ry*_) can be determined by the relationship between annealing temperature and (Eq. 2). A linear slope fit of *ln (t)*, where *t* is the time for *X*_*f*_ to reach the initial transition of 0.9%, versus *1/T* yields *E*_*ry*_*/R* (Fig. 1(b)). Thus, the value of the *E*_*ry*_ in as-deposited NT-Cu is 79 ± 8 kJ/mol.

For solid state transformations involving nucleation and growth, the Avrami model (Eq. 3) is generally applied^27^,^28^. This equation relates the recrystallization volume fraction *X*_*f*_ to time, *t*^13^,^29^.


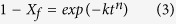


where *k* is the rate and a constant that depends on annealing temperature, nucleation rate and growth rate. Taking the logarithm of Eq. 3 gives the classical Johnson-Mehl-Avrami-Kolmogorov (JMAK) model^27^,^28^:


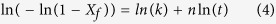


where *n* is the JMAK exponent. The values of *n* reflect the nucleation and growth morphology^29^. Values of *n* and *k* can be derived from the slope and intercept of a *ln (−ln (1−X*_*f*_)) versus *ln t* plot. Data points indicating the recrystallization stage in Fig. 1(a) were replotted in Fig. 1(c). The *{ln (−ln (1−X*_*f*_*))- ln (t)}* plot was close to a straight line. The summary of the kinetic parameters for recrystallization is presented in Table 1. The *n* values in Table 1 decrease slightly with annealing temperatures, but all are less than 1.

In contrast to *n*, Table 1 shows that *k* values increase with annealing temperature. The temperature dependence of *k* can be expressed by the Arrhenius Eq. 5:


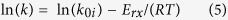


where *k*_*0i*_ is an effective frequency factor under isothermal conditions and *E*_*rx*_ the apparent activation energy (kJ/mol) for recrystallization. The slope of a linear fit of *ln (k)* versus *1/T* yields *E*_*rx*_*/R*. This indicates that the reaction rate is only affected by temperatures. The frequency factor *k*_*0i*_ is scattered around (1.22 ± 0.2) × 10^12^ based on the intercept in Fig. 1(d). The value of the apparent activation energy *E*_*rx*_ was 136 ± 3 kJ/mol for recrystallization, estimated from the slope in Fig. 1(d). Using the derived *E*_*rx*_ and *k*_*0i*_ values, Eq. 3 was expressed as:


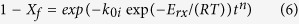


The *1* − *X*_*f*_ vs *t* data in Fig. 1(a) was fitted in accordance with Eq. 6. The solid black line in Fig. 1(a) detailed the curved fits that had been obtained at each temperature. The fitting curves matched well with the experimental data. Therefore, using Eq. 6, *E*_*rx*_ and *k*_*0i*_ within their valid temperature range, we calculated the softening time at a given temperature. For example, at 100 °C, it would take at least 36 years to soften our as-deposited materials to half the original hardness and at room temperature, and the hardness would not show any evident change for hundreds of years. This result indicates that although thermodynamically, NT Cu is a metastable material, the kinetics of restoration is sluggish.

### Thermal behavior during non-isothermal DSC

**Figure 2:**
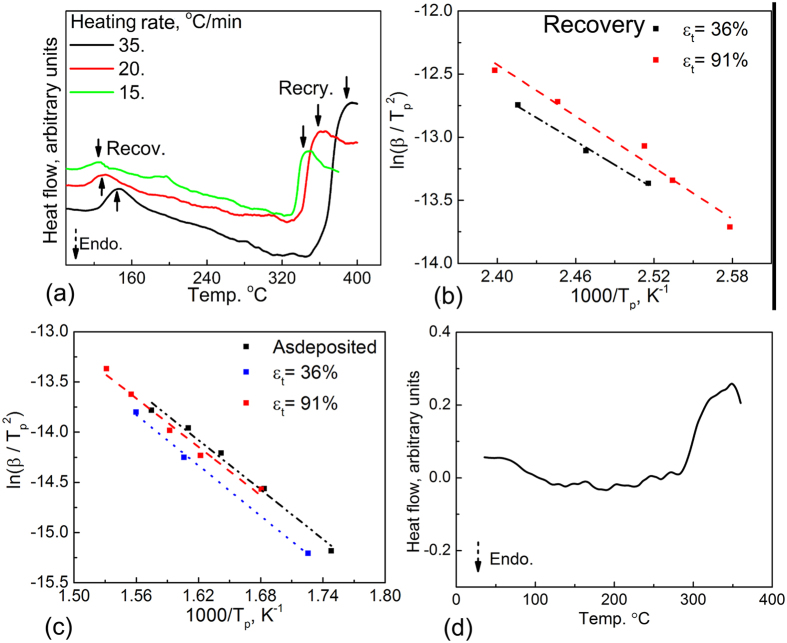
(**a**) DSC curves showing that the peak positions shift to higher temperature with an increasing heating rate *β* (15, 20, and 35 °C/min) in NT Cu deformed to 36% strain. (**b**) Kissinger plot of the temperature shift for recovery as a function of heating rates (the estimated activation energy is 56 ± 5 kJ/mol from these plots). (**c**) Kissinger plot of the temperature shift for recrystallization in as-deposited and deformed NT Cu, respectively (the estimated activation energy is 68 ± 4 kJ/mol from these plots. *ε*_*t*_ stands for true strain, *T*_*p*_ is the temperature at peak position on DSC curve. (**d**) A typical DSC curve on as-deposited NT Cu.

Assuming that the transformation rate (namely, *dX*_*f*_*/dt*) reached a maximum at the temperature *T*_*p*_ where DSC curve displayed the peak, Kissinger reached a relation between the temperature *T*_*p*_ and heating rate *β*^30^


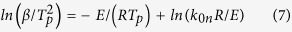


where *k*_*0n*_ is an effective frequency factor under non-isothermal condition and *E* is the activation energy. Peak temperatures were determined from the DSC curves, as shown in Fig. 2 (a). Following Kissinger’s analysis, the slope of a linear fit of *ln* (*β*/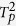) versus *1/T*_*p*_ yields *E/R*, see Fig. 2(b,c). The values of *E* was determined as 56 ± 5 kJ/mol for recovery in deformed NT Cu (Fig. 2(b)) and 68 ± 4 kJ/mol for recrystallization in both as-deposited and deformed NT Cu (Fig. 2(c)) and these activation energies appear independent of deformation strain in deformed NT Cu (see Supplementary S2). Using *E*, the relation between heating rate *β* and peak temperature *T*_*p*_ can be derived as


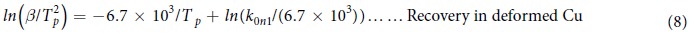



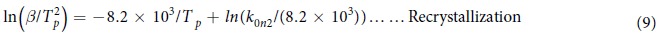


The values of the pre-exponential factors *k*_*0n1*_ and *k*_*0n2*_ for recovery and recrystallization in as-deposited and deformed NT Cu were shown in Table 2. The corresponding recrystallization factor *k*_*0n2*_ of as-deposited NT Cu was close to that of 36% deformed NT Cu, but slightly higher than that of 91% deformed NT Cu. For recovery, however, the *k*_*0n1*_ of 36% deformed NT Cu was one order of magnitude lower than that of 91% deformed NT Cu. This means that in as-deposited NT Cu the recrystallization rate is not necessarily lower than as-deformed samples once the materials reach the recrystallization temperature. In deformed NT Cu, the recovery rate became high with deformation strain because of the stored energy of high densities of primary dislocations^22^. With the known parameters of *k*_*0n*_ and *E*, the peak position for recovery or recrystallization at certain heating rates in NT Cu can be determined using Eqs 8 and 9.

In as-deposited NT Cu, only the recrystallization peak was clearly visible on the DSC curves (Fig. 2(d)). This observation is consistent with very careful calorimetric work on pure copper^19^,^20^,^21^,^22^,^31^. Our previous research has shown clean NT structure and few dislocations in as-deposited NT Cu^5^. The NT Cu described in this paper is characterized by dislocations of [112]/6 type at columnar boundaries. The maximum density of these dislocations is small and was estimated at 1.2 × 10^9^ mm^−2^ (see Supplementary S6). This density is very close to the typical dislocation density in annealed copper^32^ (10^9^ mm^−2^). The energy of dislocations is proportional to the square of the burgers vector, *b*^2^, and can be described by 
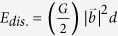
, where *E*_*dis*_ is the dislocation associated energy, *G* is the shear modulus (42 GPa) and *d* is the dislocation length. Therefore, the energy of the [112]/6 dislocations per molar volume is about 4.5 J (Copper lattice constant, *a* = 0.3615 nm) and is close to the baseline noise in DSC curves. Therefore, the recovery peak was not clearly visible and could only be studied by isothermal method, as described in the last section. Nevertheless, isothermal and non-isothermal experiments are two different perspectives, but complementary methods for studying the stability of the materials.

### Microstructure characterization

**Figure 3:**
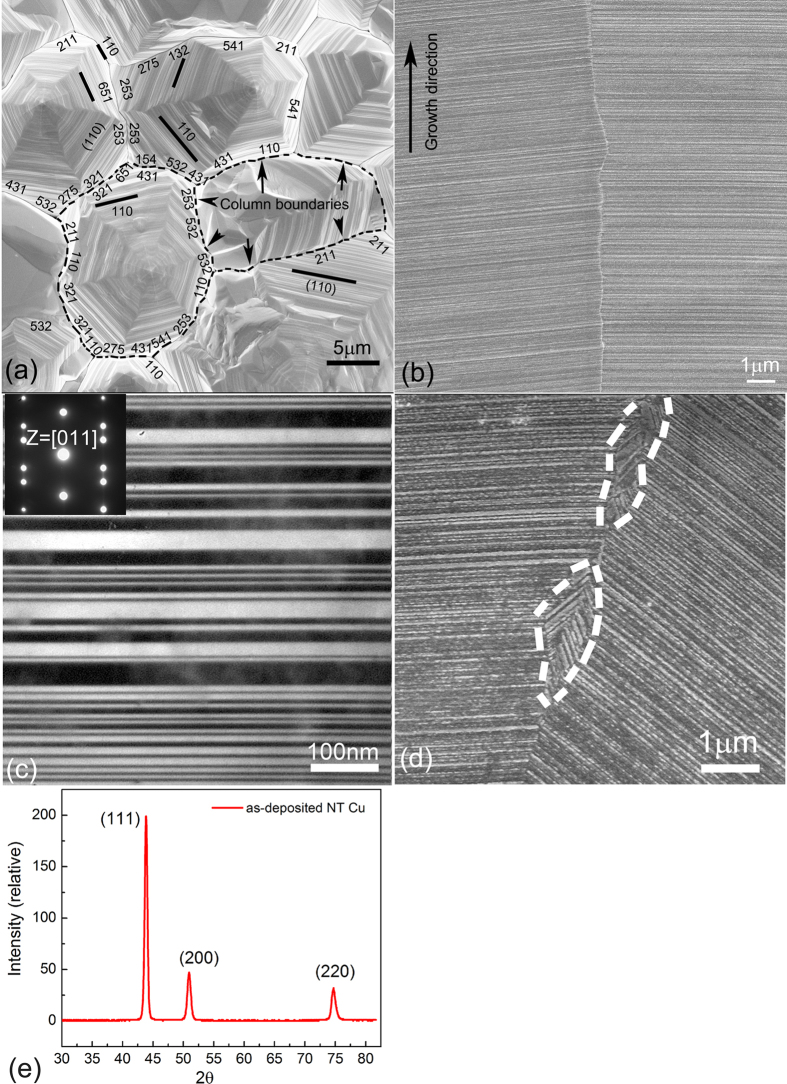
As-deposited NT Cu: (**a**) SEM image on the surface morphology. The hexagons imply the {111} crystal planes. The hexagon columns are connected by CLBs which are identified as mostly high-angle boundaries (based on ~100 SEM images, and one image includes at least 20 CLBs). Some of the boundaries are indexed. (**b**) SEM image on cross-section showing that each column is filled with a high density of {111} twins. Twin planes in adjacent columns are parallel to each other. (**c**) TEM image showing nano-scale twins. (**d**) SEM image showing twin planes in adjacent columns are inclined, and few non-NT features at CLBs. (**e**) XRD pattern.

Plane view SEM micrographs of as-deposited NT Cu foils show numerous equiaxed columnar grains. The column size varies from 1 μm to 12 μm. These columns have an overall hexagonal structure (Fig. 3(a)). Another pronounced feature is the zigzag shaped CLBs that separate columnar grains. Most CLBs in our NT Cu were identified as HABs (Fig. 3(a)), these HABs are close to {541}, {275}, {321} and {211} crystal planes when viewing from <111>. Cross-section SEM observation (Fig. 3(b)) shows that the columns are filled with a high density of {111} growth twins. In each column, twin planes are parallel to each other. Figure 3(c) exhibits twin spacing in a range of 2~50 nm, and TBs are straight and clean. The correspondent select area diffraction pattern (an inset in Fig. 3(c)) shows that most of the diffraction spots appear to be elongated in the <111> direction due to the overlapping of the streaks with primary diffraction spots, which confirms the high density of TBs. Twin planes in adjacent columns are sometimes inclined to each other, Fig. 3(d). The XRD pattern (Fig. 3(e)) indicates that the intensity ratio of reflection peaks (111) over (200) is about 4.23, and the ratio is around 2.16 in the random oriented powder pattern. This observation is slightly different with previous strong {111} texture results, where the twin planes are most perpendicular to the growth direction^6^,^33^. The texture appears weaker in current NT Cu.

**Figure 4:**
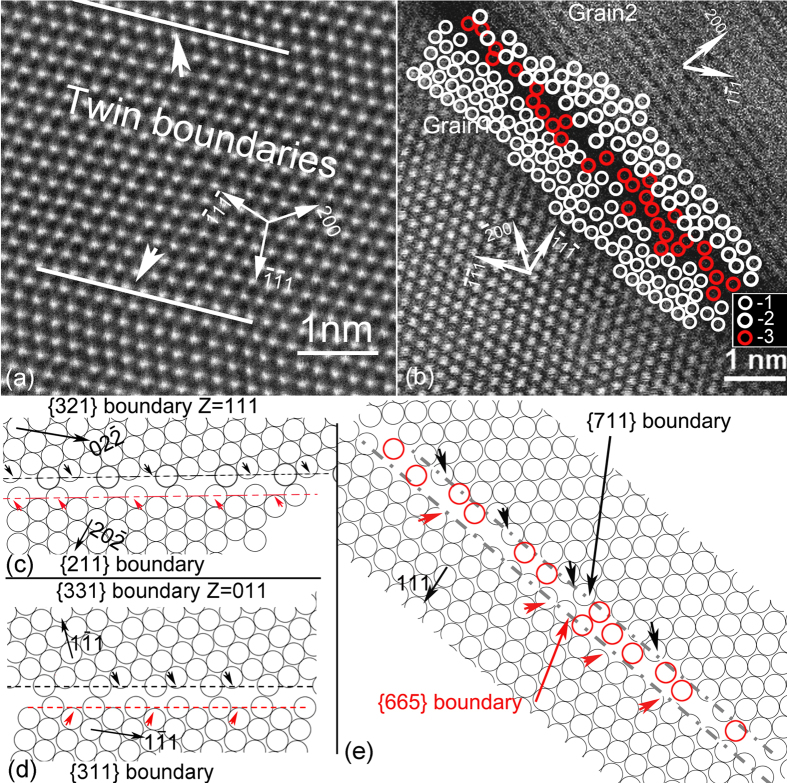
(**a**) Z-contrast image showing that two TBs in as-deposited NT Cu are straight, perfectly coherent and free of impurities at both TBs and twin interior. (**b**) Z-contrast image showing the disorder and loose columnar boundary structure. In the (**b**) inset, −1, −2 and −3 three types of hollow circles represent atoms position in grain 1, grain 2 and possible locations within boundary, respectively. The rotation axis of grain 2 is close to <110>. Based on the plane angle between the boundary with {111} plane in grain 1 and with {200} plane in grain 2, the projected boundaries with lowest miller index were estimated to be {665} in grain 1 and {711} in grain 2, respectively. (**c–e**) are schematic diagrams for estimation of the atomic densities in projected {321}//{211}(rotation axis = <
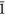
11>), {331}//{113} (rotation axis = <
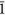
10>) and {665}//{117} (rotation axis = <
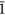
10>) interfaces, respectively. The red circles in (e) mean possible atoms. The lines with black and red arrows indicate projected {321}//{211}, {331}//{113}, and {117}//{665} boundaries, respectively. The atom positions in schematic Fig. 4(d) (Z = 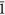
10) are close to the experimental results with relatively low indexed Miller indexed boundaries, which are projected {331}- and {113}-boundaries. The atomic positions in the grains in (e) are even closest to those in experimental image (Fig. 4(b)).

In the STEM image (Fig. 4(a)), each white dot is directly related to an atomic column position^34^. TBs are straight and Cu atoms at TBs are clear, orderly and well-arranged. The intensities resulting from the atomic column at the twin boundary and other area are uniform and identical. In other words, impurity atom segregation was not detected at TBs (based on ~30 analyzed images).

At CLBs, atoms have a disorderly and loose arrangement (Fig.4(b)). This differs from the interior orderly arrangement. In most cases the formation energy for vacancy in CLB is lower than in bulk^35^,^36^, thus vacancy clusters are usually observed in the vicinity of CLBs. To estimate the possible vacancy concentration along HABs, we assessed the atomic packing at the twist boundaries of {321}//{211}, {113}//{331}, and {665}//{117} (Fig. 4(c~e)). The atomic positions in Fig. 4(d) (Z = 011) are close to the experimental results with relatively low indexed Miller indexed boundaries. The atomic positions in Fig. 4(e) (Z = 011) is even closer to those in experimental image (Fig. 4(b)). We estimated the atomic packing density in these boundaries to be 61%, 56% and 55~51%, about one-third sparser than in the interior (80% and 90% for <111> or <110> zone axis). The atom density at CLBs in our experimental results (Fig. 4(b)) was about 63% of that of the interior. The density is close to that projected by lower Miller indexed planes ({331}//{113}) but slightly higher than {665}//{117}. Therefore, we conclude that, for CLBs with higher Miller index planes than {321}//{211} and {311}//{331}, the vacancy concentration reaches a saturation. These locations provide the source for vacancies.

**Figure 5:**
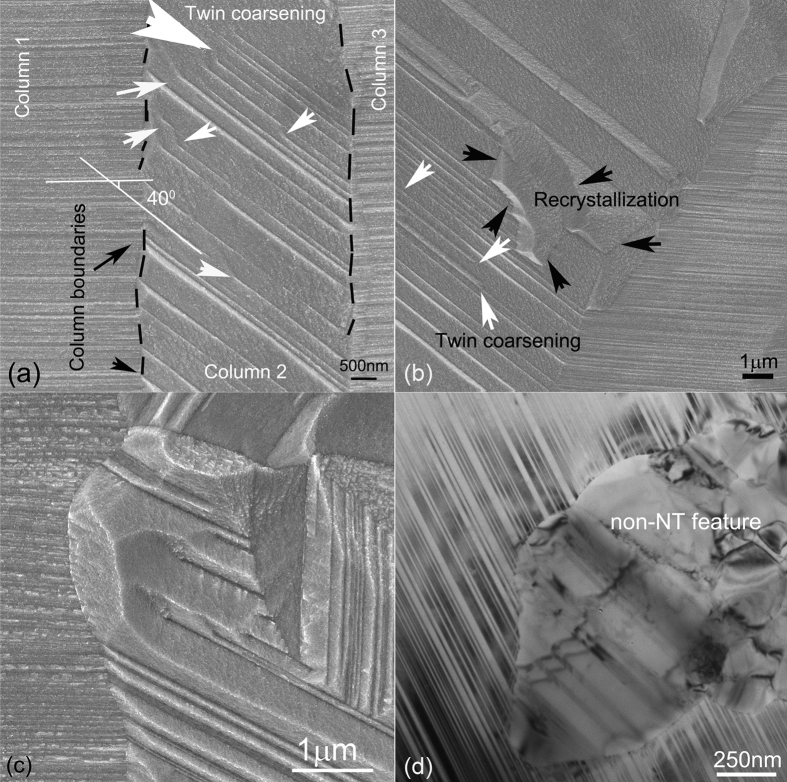
Microstructure evolution in NT Cu after 210 °C annealing for 30 minutes. (**a**) SEM image showing that twin coarsening prefers to start in the column which is inclined to the foils growth direction when the misorientation between adjacent column grains is large. The small white arrows indicate twins coarsening. The large white arrow implies twins withdraw. (**b**) SEM image showing initiation of recrystallization from the columnar boundary and new grains grow in the area swept by twin coarsening (**c**) SEM image showing the growth of non-NT features into NTs. (**d**) TEM bright field image showing non-NT feature invades into NT area before twin coarsening.

During annealing, a net force at an individual TJ sometimes results in emission of dislocation^9^. The emission of partial dislocations was the consequence of local atom shuffling events and stress-assisted free volume migration in the boundaries^37^. Consequently, vacancy-assisted structural and twin coarsening proceeds via the motion of Shockley partial dislocations along a {111} plane from one TJ on the CLBs, to another TJ on another CLBs. In Fig. 5(a) a black dashed line represents CLBs. The trace of {111} planes in column 1 are approximately parallel to the ones in column 3, and have a 40° incline angle with the ones in column 2. The region pointed out by the small white arrows in Fig. 5(a) exhibits the twin coarsening in column 2 after annealing at 210 °C for 30 minutes. The twin spacing remains very fine in both columns 1 and 3. In some regions (e.g. the region indicated by the black arrows in Fig. 5(b)), recrystallization starts. The recrystallization initiates from the columnar boundary and is growing toward the coarsening region, although the twins in neighboring columns are still very fine. Observation on Fig. 5(a,b) suggests that the twin coarsening and recrystallization are selective^38^, i.e., GB mobility depends on misorientation of the adjacent grains.

Besides twin-coarsening induced recovery and recrystallization, another recrystallization phenomenon without twin-coarsening was also observed in certain locations associated with few non-NT features (Fig. 3(d)). These non-NT features are comprised of large twins (i.e. twin spacing greater than 100 nm) and random boundaries. Random boundaries are usually incoherent, and they readily grow by directly intruding into the nanotwin matrix before nanotwin coarsening occurs (Fig. 5(c,d)). These observations in Figs 3, 4, 5, and Supplementary S4, S5 and Figs S3, S4 imply that HABs have the potential to reduce stability in NT materials.

**Figure 6:**
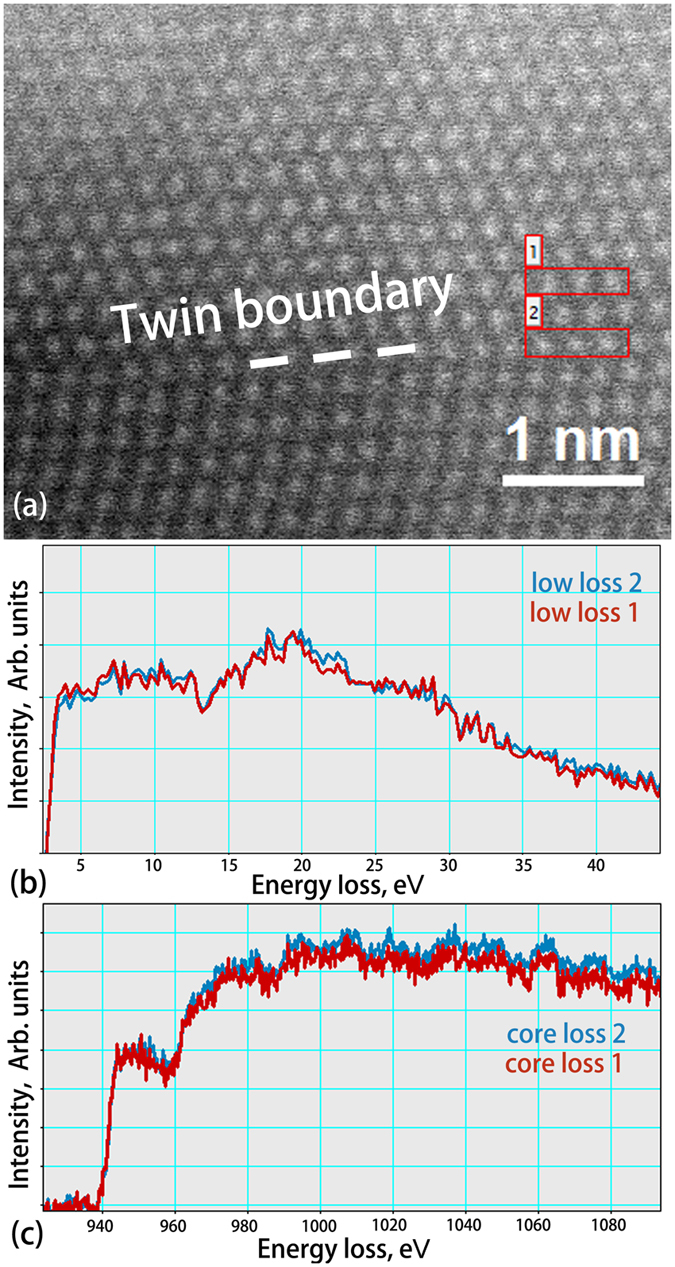
(**a**) Atomic resolution STEM-EELS survey image. The dash line indicates at atomic scale the twin boundary location. Large numbers of EELS spectra were taken for comparison. Supplementary Fig. S5 shows a fast scanned STEM image in the same area. The framed areas labeled 1 and 2 in (**a**) are the regions where the spectra were extracted for showing in (**b,c**). (**b**) low-loss spectra of ~22 nm-thick NT Cu at TB (area 2) and interior (area 1) up to energy loss of 40 eV with zero loss extracted. (**c**) L3 edge spectra from NT Cu at TB (area 2) and interior (area 1) extending ~150 eV above threshold with de-convolved and background stripped.

Electron energy loss spectroscopy (EELS) is capable of giving both structural and chemical information about a solid with an atomic spatial resolution. In NT Cu, the bonding structure and chemical composition at TBs and interior were probed by comparing the plasmon resonance in the low-loss region, Fig. 6(b), and L3 edges (representing excitation of the Cu 2p^3/2^ electron) in the core-loss region, Fig. 6(c). The EELS analyses of atomic columns at both TBs and twin interior showed the same shape of plasmon peaks, implying that the inelastic electron scattering or conductivity was the same since plasmon excitation arose from the interaction between incident electron and outer-shell electrons^39^. EELS data also revealed no difference in the L3 edge position suggesting that the Fermi level was the same at the TBs and interior because the inner-shell excitation implied transitions of the core electrons to the empty state above the Fermi Level^39^. This indicated that at room temperature, the scattering of the electrons by the twin boundary atoms was below the detectability of EELS and TBs have limited influence on conductivity.

## Discussion

To interpret the meaning of the low value of *n* in Table 1 during recovery and recrystallization, it is essential to understand the JMAK model. This model is precise under certain assumptions: (1) nucleation is uniform but random; (2) the form of new particles or grain is spherical, etc^29^. Our experimental values of *n* are generally smaller than 1 and different from the theoretical *n* values analyzed using the linear growth model because our experimental condition does not meet the above assumptions. We divide the JMAK exponent into three parameters as: *n* = *a* + *b/c*, where *a* is the nucleation index; b is the dimensionality of growth; c is either 1 for linear or 2 for parabolic growth^40^. If pre-existing nuclei are present or the site is saturated, *a* = 0; if the nucleation rate is time independent, *a* = *1*. The growth dimensionality *b* is 1, 2, or 3 for one-, two-, or three-dimensional growth respectively. For one-dimensional linear growth, if *a* = *0*, then *n* = *1*; if *a* = *1*, then *n* = *2*, thus, *1* ≤ *n* ≤ *2*. For one-dimensional parabolic growth, however, *0.5* ≤ *n* ≤ *1.5*. Because the values of *n* at our study are between 0.3 and 0.8, we assume c equal to 2, indicating that the growth is likely one-dimensional parabolic at most temperatures. Inhomogeneous nucleation and larger growth rate anisotropy reduce the JMAK exponent *n* significantly^41^. SEM observations did show that both twin coarsening and growth were extremely constrained. Twins coarsen when the Shockley partials move along 111 planes forming steps (Fig. 5(b) small white arrows) or when a group of twins withdraw cooperatively forming long ITBs (Fig. 5(a) large white arrow). We mainly observed the coarsening that resulted from partial motion. With pre-existing nucleation sites, *n* depends mainly on growth geometry. The Shockley partials move only in one direction between two HABs, resulting in the value of *n* close to 0.5.

In our NT Cu, the values of activation energy for recovery were found to be 79 ± 8 kJ/mol from the JMAK equation and 56 ± 5 kJ/mol from the Kissinger equation. The values of activation energy for recrystallization were around 136 ± 3 kJ/mol from the JMAK equation and 68 ± 4 kJ/mol from the Kissinger equation. Apparent activation energy values for recovery and recrystallization measured by isothermal annealing are 10% and 50% higher than the ones evaluated by non-isothermal DSC. Similar discrepancy between the isothermal and non-isothermal measurements was also observed by others^42^. For example, for a fluoride glass studied by Bansal *et al*., the activation energy determined by isothermal DSC was found to be about 18% higher than that determined by the Kissinger equation^42^. Not many comparisons are available for the kinetic parameters of recrystallization determined by different thermal analysis techniques. The activation energy difference calculated from JMAK and Kissinger equations may be caused by the difference in kinetics in isothermal and non-isothermal processes. During the isothermal process, the activation energy derived from the JMAK equation is based on the measurement of the isothermal transformation rate; while the activation energy derived from the Kissinger equation is based on the measurement of the extent of transformation as a function of both time and temperature during the non-isothermal process. Similar to the relationship between the time-temperature transformation (TTT) and continuous cooling transformation (CCT) diagrams, the activation energy from the JMAK equation is better to describe the isothermal phase transformation, whereas the Kissinger equation is more adapted to the continuous process.

There are several crucial factors accounting for the activation energy of recovery and recrystallization: purity, TJs, vacancies and high-angle boundaries. These factors were addressed individually in the following paragraphs. The drag-effect by impurity particles^25^ on GB motion is especially important in nanocrystalline systems, where the grain microstructure is highly unstable owing to the large GB area per unit volume. Even small amounts of impurities drastically reduce the velocity of GB motion. For instance, a small sulphur content increase from 0.11 to 1.1 wt ppm reduced GB self-diffusivity by a factor of about 15 in pure Cu^43^. Nanotwins in sputter-deposited Cu remained stable at temperature of 800 °C due to a Zener drag force by 0.5 at% Fe precipitates at the grain boundaries^4^. Lucke *et al*. attributed the drag-effect of impurities to their cooperative motion with the boundary^44^,^45^. Gas and co-workers^46^ demonstrated that the impact of the impurity segregation is dependent on the impurity-impurity and impurity-matrix bonds. Gottstein and Shvindlerman^15^,^47^ reported that in Cu the second-phase particles with drag-effect were estimated to be 15–50 nm, below which the GB will repel and not wet the particles^15^.

Inductively coupled plasma mass spectrometry (ICP-MS) analysis revealed in our NT Cu^6^, that elements with the highest concentration (i.e., Ba, Pb and Cr) were at the ppm level. Among those three elements, only Ba has a strong bond with Cu and formed compounds with Cu. Therefore Ba may have strong influence on GB migrations. Our Z-contrast image in Fig. 4(b) showed that the thickness of columnar boundary was about 0.5 nm. The estimated impurity content at the columnar boundary is about 4.56 wt.% if we assume the 2.28 ppm Ba content is completely segregated at columnar boundary and the column size is about 10 μm. The impurity occupies less than 5% of the grain boundaries. Combined with the above analysis, we concluded that the impurity impact on GB migration was negligible because of the low segregated impurity content and the small size. This is one of the reasons that our NT Cu softened at much lower temperatures than materials made by other researchers^48^.

TJs along with CLBs are the main microstructural elements of NT Cu. GB property strongly depends on GB crystallography. The energy of the TJ lines and their line tension is significant in nanocrystal materials^10^,^15^,^47^,^49^. In nanocrystalline Cu, the triple line energy was reported larger than the GB excess energy, The excess energy of boundary junctions along with the GB energy constitutes the driving force for grain growth^10^. Therefore, in NT Cu, TJs contribution to the nanotwin coarsening is believed to be predominant.

As addressed earlier the vacancy concentration would be even higher at the vicinity of CLBs with higher Miller index planes. The kinetics of vacancy-mediated processes is proportional to the probability of vacancy diffusion^50^,^51^, 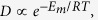
where *E*_*m*_ is the vacancy migration energy, and *T* is the absolute temperature. Overhauser estimated activation energy for vacancy motion (*E*_*vm*_) about 65 kJ/mol^50^ in irradiated Cu. Kalu reported the activation energy for vacancy migration to be 20 kJ/mol in drawn oxygen-free high thermal conductivity Cu^52^. In electrodeposited Cu, recrystallization was reported to occur at temperatures as low as ambience, and the recrystallization activity energy was estimated to be 86 kJ/mol^17^. Gangulee^53^ determined the activation energy for recovery (28 kJ/mol) and recrystallization (57 kJ/mol), in electroplated Cu. The activation energy in the above references was determined under a static process, same as the activation energy calculated from JMAK equation in our current work. The activation energy for recovery (79 ± 8 kJ/mol) and recrystallization (136 ± 3 kJ/mol) are lower than 200 kJ/mol, substitutional diffusion of Cu^54^. Considering the activation energy of vacancy migration (20~65 kJ/mol)^50^,^52^ and boundary migration (*E*_*b*_ = 104 kJ/mol)^13^, we conclude that the recovery process is controlled by vacancy migration (which contributes to dislocation motion), while the recrystallization process is dictated by the motion of TJs and high angle boundaries.

The CTB structure showed in Figs 3(c) and 4(a), Supplementary S3 and Fig. S2(a) is obviously different from the one in Wang’s report, where the as-grown CTBs in NT copper were inherently defective with curvature and kink-like steps and these steps were ~1–5 nm high ITBs^8^. Comparatively, in our samples, Cu atoms in the boundary were all in a straight line and fit perfect into both adjacent grains. TBs were parallel to the twinning plane and no steps were observed in a series of Z-contrast images (Fig. 4(a) and Supplementary Fig. S2(a)). In other words, for the average 10 μm column, nearly 4.61 × 10^4^ atoms were strictly aligned in the twin boundary. We believed that most of TBs were perfectly coherent in our NT Cu according to the original definition in the text book^54^. Consequently, the contribution of TB incoherency to activation energy, twin coarsening and recrystallization can be considered minor. Higher thermal energy (or temperature) is required in order to generate enough mobile Shockley partial dislocations from CLBs. Introduction of ITBs renders NT Cu unstable, as we have shown in our previous experimental work, where the influence of ITBs on microstructure and mechanical properties has been investigated in details^5^,^6^. Partial dislocations were also reported to be generated during the twin formation as previously reported^7^,^8^. Strain-induced ITBs were demonstrated to be highly mobile because they inherently contained Shockley partial dislocations. The high mobility of the partial dislocations with high density resulted in subsequent twin coarsening, grain growth or recrystallization at relatively low temperatures. Therefore, our previous observations and analysis are complementary to the simulation results of Wang *et al*.^7^,^8^.

## Summary

NT Cu is a metastable crystal. The NT Cu studied in this work is characterized by high angle columnar boundaries, i.e., HABs that are about 0.5 nm-thick. TJs occur where HABs meet NT boundaries. The movement of boundary in single direction is driven by the combined high energy of TJs and HABs. The NT samples in this study are characterized by low concentration impurity throughout (<2.6 ppm), and high vacancy concentration at columnar HABs, this facilitating boundary migration, without impurity dragging. The mono-directional boundary motion that occurred in our samples caused by TJs and HABs may have been the cause of the high degree of anisotropy in twin coarsening that we observed. It may also have encouraged grain growth from pre-existing nuclei, leading to JMAK exponents for recovery and recrystallization below 1, which indicates stable NT structure at temperatures below 100 °C. The thermal stability of NT Cu is dictated by four factors: 1) the presence of TJs, 2) the concentration of vacancies, 3) the concentration of impurities, and 4) the density of HABs. Optimization of these four microstructural features is critical for fabrication of stable nano-structured materials.

## Methods

### Sample preparation

High purity NT Cu (impurity content < 2.6 ppm) foils (50 × 10 × 0.1~0.2 mm^3^ in size) with a high density (96%) of CTBs were synthesized by a pulsed electro-deposition technique. A copper sheet with of 99.99% purity was used as the soluble anode without any additives in the solutions. The cathode substrate was a 0.1 mm thick sheet of cold-rolled MP35N alloy, which has a fcc structure and {110} out of plane texture^55^. The CuSO_4_ concentration was 28 g l^−1^ and the pH value was adjusted to 0.9 by the addition of H_2_SO_4_. Preparation details were described in ref. 33. By adjusting the electro-deposition parameters, such as the cathode current density and pulse on or off time, copper with different twin thicknesses and column sizes could be prepared. Different with the previous reported NT Cu^5^,^6^, some irregular non-NT structures were introduced into the NT Cu foils during the deposition. The deposited Cu foils were removed from the substrates after deposition. Some of the as-deposited NT Cu foils were deformed in plane-strain conditions by cold-rolling with the rolling plane perpendicular to the growth direction of the foils. Part of the deformation energy remained in the crystal lattice as stored energy. Deformation-induced dislocation density can be around 10^10^/cm^2^ 56, which can be measured by differential scanning calorimeter (DSC).

### DSC analysis

DSC is used to measure stored energy that is related directly to the microstructure evolution such as recrystallization and grain growth^22^,^57^. The thermal stability of NT Cu was assessed by a Shimadzu DSC-60 during annealing. The DSC instrument had been calibrated with pure Indium (99.999%) and Tin (99.999%) standards. For Indium, the referenced enthalpy is 28.5 ± 0.2 J/g; our measured value was 28.6 ± 0.3 J/g. The systematic error is 0.9%. The melting point (or peak temperature *T*_*p*_) error is 0.14 °C for indium (*Tp* = 156.63 °C), and 1.8 °C for Tin (*Tp* = 231.9 °C). The peak temperature error is less than 0.8%. The measured heating rate for 10, 15, 20, 35 °C/min are 9.6, 14.6, 19.4, 34.6 °C/min, respectively. The linear fitting of the measured heating rate with the command heating rate has a slope of 1.0004 ± 0.0044. The error is 0.4%. In the DSC analysis, samples were sealed in aluminum pans and heated from room temperature to above recrystallization temperature at a number of heating rates between 5~40 K/min in an argon atmosphere. At each heating rate, a fully recrystallized copper sample was scanned to provide a baseline reference for each DSC run so that the heat evolution detected was only from the restoration processes taking place in each sample. For deformed NT Cu, DSC tests were conducted immediately after the rolling deformation.

### Hardness tests

Isothermal stability of NT Cu was assessed by hardness testing. NT Cu samples were heated rapidly to the desired temperatures and held for different durations. Micro-hardness tests were performed by a Tukon 2100 microhardness tester with a Vickers diamond pyramid indenter on the substrate side of as-deposited, rolled and annealed foils. This complies with ASTM standard E384^58^. The hardness of rolled samples was measured immediately after rolling. The tester was calibrated before every experiment. The scattering of the hardness value from the standard sample is 1.2%, which was attributed to systematic error. To select the proper load, hardness was done with loads of 50, 100, and 300 g on typical samples, and the corresponding hardness numbers were found within 3% scattering. Therefore, load difference resulted in no evident difference in hardness value readings. The indents were wider than 20 μm, measured diagonally, in accordance with the ASTM standard^59^. Further indent examinations revealed that the indent depths were only 2, 3 or 5.5 μm. Therefore, the foil thickness was at least ten times the depth of the indentations; once again, this complies with the ASTM standard^60^. No marks could be observed on the surface to the opposite to the indentation. To confine and minimize the deformation zone within the foil under indentation without losing accuracy, a 50 g load was selected and used for generating the data reported in this paper. The time duration was 10 seconds. Our scanning electronic microscopy (SEM) observations at the cross-section of indents revealed that the visible deformation zone under the indentation tip was less than 1 μm, which further confirmed that 50 g was a proper load for measuring hardness, not so high as to penetrate the foils but not so low to distort the mechanical properties. Six to ten tests were conducted for each measurement.

### Microstructure characterization

Transmission electron microscopy (TEM) has been an indispensable tool in materials research for obtaining valuable information about material structure and properties. To avoid the damage resulting from sample preparations, we developed a new cross-section preparation method as shown in ref. 33. Using the new method, the cross-sectional samples for the TEM examinations were prepared by twin jet electro-polishing with a solution of 33% phosphoric acid in water at 5.5 V and 24 °C.

Both the transmission electron microscopy (TEM, JOEL-2011) and sub-angstrom TEM/STEM (scanning transmission electron microscope, JEM-ARM200cF) observations were operated at 200 kV. The probe current used for acquiring the high angle annular dark-field HAADF images was 39.4 pA. The condenser lens aperture size was 40 μm. The camera length was 8 cm/6 cm and the collection angle was 76–174.6 mrad/90–174.6 mrad. One and two-dimensional EELS analysis was performed with a Gatan Dual EELS^TM^. EELS data were acquired in a STEM mode with a probe size of ~0.2 nm and probe current of 324 pA, convergence and collection semi angles of approximately 32 and 92 mrads, respectively. The EDS analysis was performed using an EDAX instrument attached to the ARM microscope. Spectra line scans as well as chemical maps for the various elements were obtained using the EDAX Genesis software.

The plan-view and cross-section morphologies of NT Cu samples were characterized by scanning electron microscopy (SEM, Zeiss 1540) operated at 7 kV. Samples for cross-sectional examinations by the SEM were first cold-mounted, then mechanically ground and polished and finally etched for 1 min in a fresh solution of 20 ml NH_4_OH and 20 ml 3% H_2_O_2_.

## Additional Information

**How to cite this article**: Niu, R. *et al*. Influence of grain boundary characteristics on thermal stability in nanotwinned copper. *Sci. Rep.* **6**, 31410; doi: 10.1038/srep31410 (2016).

## Supplementary Material

Supplementary Information

## Figures and Tables

**Table 1:** Values for the recrystallization kinetic parameters from isothermal analysis.

Temperature (°C)	Recrystallization	
	*k*	*n*
160	(5.4 ± 0.5)E-05	0.75 ± 0.03
210	(2.4 ± 0.3)E-03	0.56 ± 0.03
230	(9.2 ± 0.3)E-03	0.56 ± 0.02
290	(2.9 ± 0.2)E-01	0.32 ± 0.02

**Table 2:** Values of pre-exponential factor for recovery and recrystallization from DSC results.

True strain (%)	Recovery ***k***_*0n1*_, x10^3^	Recrystallization ***k***_*0n2*_, x10^3^
As-deposited		3.8 ± 0.5
36	66.9 ± 6.8	3.9 ± 0.4
91	302.9 ± 12.9	2.8 ± 0.3
