# Supplementary material for: Influence of grain boundary characteristics on thermal stability in nanotwinned copper
Source: Sci Rep. 2016 Aug 12;6:31410. doi: 10.1038/srep31410 (PMC4981844; doi:10.1038/srep31410)
Supplement: Supplementary Information [file srep31410-s1.pdf]

Influence of grain boundary characteristics on thermal stability in nanotwinned copper

Supplementary Materials

Rongmei Niu[1], Ke Han[1*], Yi-feng Su[1], Tiglet Besara[1], Theo M Siegrist[1], and Xiaowei Zuo[2]

1) National High Magnetic Field Laboratory, 1800 E. Paul Dirac Drive, Tallahassee, FL32310

2) Key Laboratory of Electromagnetic Processing of Materials (Ministry of Education), Northeastern University, Shenyang 110004, China

*Corresponding author at: han@magnet.fsu.edu. Tel.: +1 850 6446746, fax: +1 850 6440867

Supplementary :

Supplementary S1. Calculation of activation energy in isothermal experimental condition and activation energy comparison

Even though recovery detailed mechanisms are not the focus of this article, we compared the activation energy values of recovery and recrystallization at different stages (Fig. S1).

To calculate the activation energy of recovery, we need to read the annealing time for X_f from Fig. 1(a). Those values can be either experimental or extrapolated data. At X_f =36%, we have one experimental datum at 230 ˚C and we extrapolated the other data for other temperatures. From Fig. 1(a) we can clearly see that when X_f =36%, the recrystallization occur at all the annealing temperatures. The apparent activation energy E_{rx} was evaluated at about 136±3 kJ/mol for recrystallization according to the slope in Fig. 1(d). Assuming that Eq. 2 is applicable to estimate the recrystallization activate energy, it would be 142±3 kJ/mol at X_f ~36% and slightly higher than the above value of 136±3 kJ/mol (calculated by Eq.5). If the system error was taken into account (the error bar in Fig. 1(a)), the error is ± 11 kJ/mol. The activation energy difference becomes insignificant. However, combined with the subsequent microstructure observation, we assume that the recrystallization is responsible for the softening at this stage.

We further confirmed this by the comparison of the extrapolated activation energy obtained using equations 2 and 5 (Fig. S1). By doing so, we verified that at X_f =36%, recrystallization occurred and we should use the energy obtained in equation 5. We also did similar analyses at X_f greater than 36% and the conclusion was the same, as shown in Fig. S1. On the other hand, we also did the analyses at X_f smaller than 36% (e.g. X_f =13% and 26%). The obtained activation energy value is gradually increasing with increase of X_f. This change of the activation energy

when X_f is below 36% is attributed to both 1) change of the mechanisms from vacancy annihilation to dislocation motion and their transition and 2) transition period from recovery and recrystallization. Nevertheless, the data indicate that when X_f is below 36%, recovery is dominant and when X_f is above 36%, recrystallization is dominant.

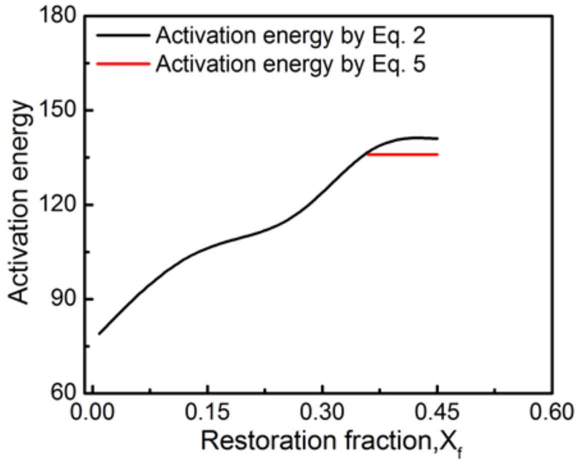

Supplementary Fig. S1. Activation energy evolution with the restoration fraction using Eq.2 and Eq.5.

Supplementary S2. Calculation of activation energy in non-isothermal experimental condition

We used the linear regression method to obtain the slopes in Kissinger plot of samples of as-deposited and deformed (Figs. 2 (b) and (c)). For recovery, the slope values (*E/R*) for recovery are -6.25±0.44 and -6.78±0.63 K for samples deformed to 36% and 91%, respectively, as shown in Table S1. We then used t-test to compare these two energy values. The estimated *t** is smaller than the critical value with 95% confidential value. This result indicates that there two values have no significant difference. We also used the similar method to analyze the slopes in other reactions and concluded that deformation strain caused almost no change in the activation energy, as shown in Table S1. Therefore, in the main text, we used the average values from those obtained in as-deposited and deformed samples to calculate the activation energy.

Supplementary Table S1. The slope difference in Figs. 2(b) and (c).

	deformation true strain	Sample mean	Standard deviation	Sample size	Test statistic,t^*	$t_{0.95}$
Recovery	36%	-6.25	0.44	3	1.265	1.943
	91%	-6.78	0.63	5		
Recrystallization	As-deposited	-8.22	0.50	5	0.475	1.943
	36%	-8.38	0.40	3		
	As-deposited	-8.22	0.50	5	0.439	1.860
	91%	-8.06	0.65	5		
	36%	-8.38	0.40	3	0.761	1.943
	91%	-8.06	0.65	5		

Supplementary S3. Identification of the atomic columns at grain boundaries

To understand the stability of the NT boundaries and CLBs, we need to identify the exact location of the atoms. To achieve this goal, we used both atomic resolution HRTEM and HAADF STEM images. In HAADF STEM images, we were able to identify most of the atomic columns at the boundaries without imaging simulation, although sometimes we used inverse fast foriour transformation to reduce the noise (Fig. S2(a)). We cannot find any steps at the coherent NT boundaries using this technique.

Using HRTEM images, we were able to readily identify the location of the boundaries, but we had difficulties to probe the single atomic column positions without doing image simulation because the FEG gun provided coherent beam and samples can be imaged at very large defocused values. Therefore, in analyzing the exact location of the atoms at the boundaries, we largely used HAADF STEM images.

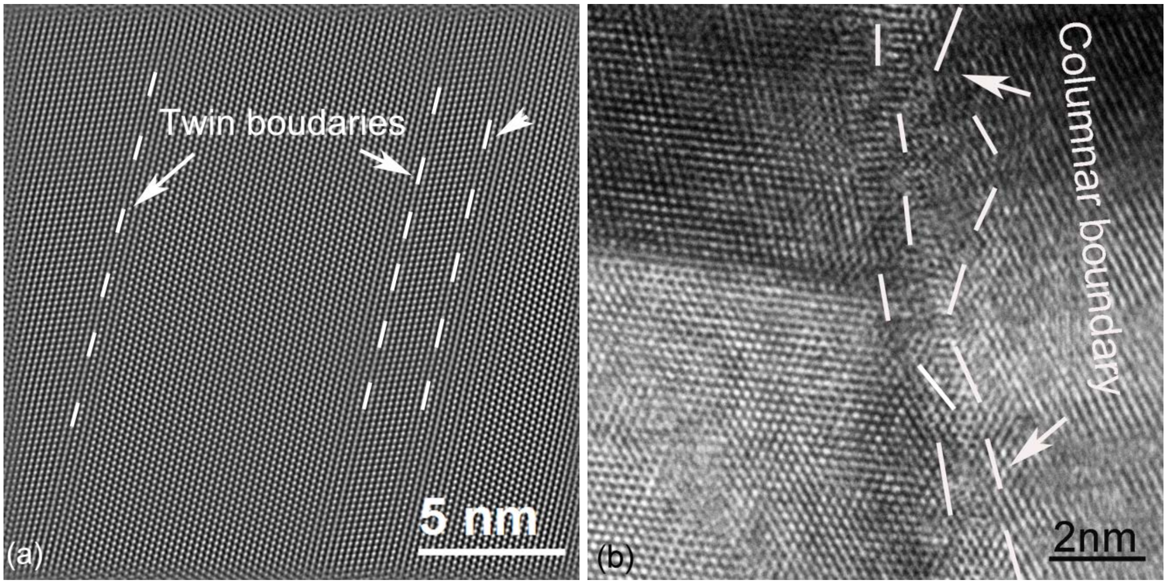

Supplementary Fig. S2. (a) Inverse fast foriour transformed Z-contrast image(STEM) showing the coherent twin boundaries in as-deposited NT Cu. Three white dash lines outline exact the location of the atomic columns at three coherent twin boundaries. (b) HRTEM image showing the columnar boundary. Due to the delocalization, the exact columnar boundary is hardly identificable, therefore, is marked by white dash lines.

Supplementary S4. Inclined twin boundary

Most of the NT boundaries have their {111} planes perpendicular to the growth direction. Therefore when samples were section perpendicular to the {111} planes, most of NT boundaries appear parallel to each other or have small angle to each other across the CLBs. In the vicinity of small numbers of CLBs, we observed the {111} planes are not parallel to each other, but inclined with respect to both CLB and each other, as shown in Figs S3 and S4.

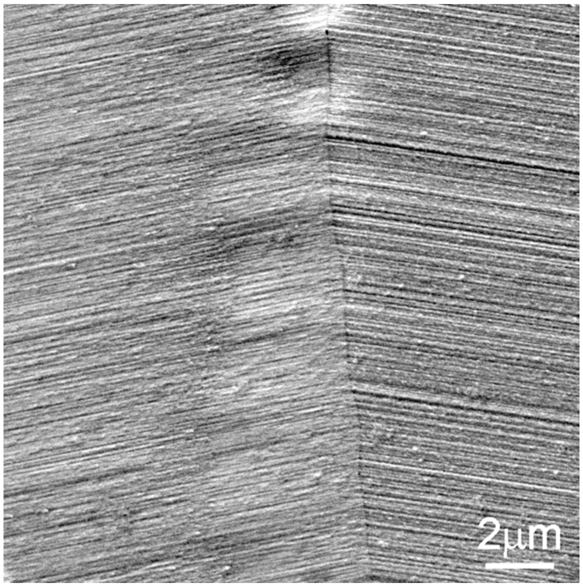

Supplementary Fig. S3. SEM image showing a column boundary inclined with respect to both groups of twin boundaries in as-deposited NT Cu.

Supplementary S5. Comparison of twin boundaries

By carefully selection the imaging location, we were able to identify the images that include NT boundaries that are perpendicular to the growth direction and NT boundaries that are inclined with respect to the growth direction. In our samples, most of coarsening started at the included NT boundaries in samples annealed at relatively short time and low temperatures (Fig. S4). In the same sample, NT is still stable in the area where NT boundaries are roughly perpendicular to the growth direction.

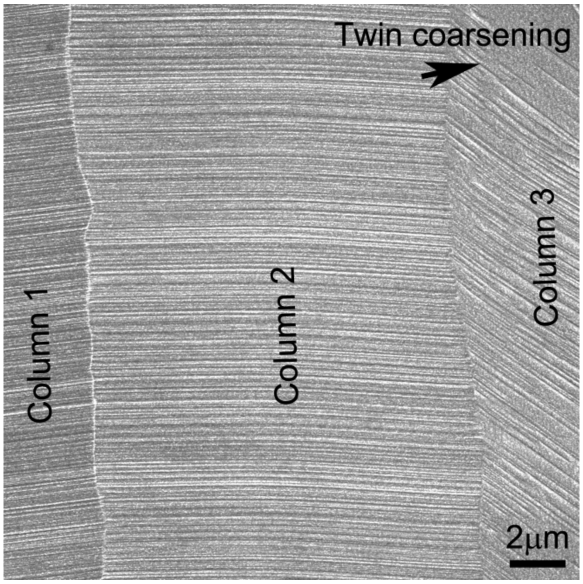

Supplementary Fig. S4. SEM image showing an annealed NT Cu: Twin coarsening and recrystallization (one example is indicated by an arrow) start in the inclined column 3. No evident coarsening and recrystallization occur in columns 1 and 2. Both columns 1 and 2 have their {111} approximately parpendicular to the growth direction.

Supplementary S6. Dislocation density estimation

In as-deposited NT Cu, few dislocations were observed within the columns. Most dislocations are at the columnar boundaries. We assume that the dislocation length equals to the column size, the maximum dislocation density (ρ) of 1.2 x 10^9 mm^{-2} was derived based on the column size (n), 111 plane spacing in Cu (d), and the foil's thickness (h).I.e

$$\rho = 2 * \frac{h}{d} * \frac{n}{n * n * h} = \frac{2}{d * n}$$

where n= 8 μm, and d= a(Cu lattice constant)/$\sqrt{3}$.

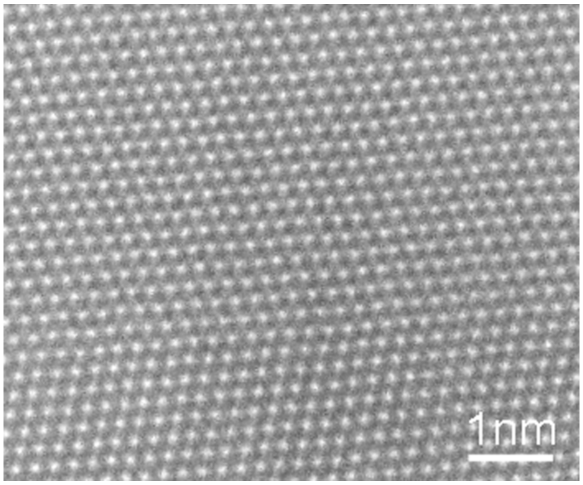

Supplementary Fig. S5. STEM image with short beam dwell time on each atom column in as-deposited NT Cu. Beam dwell time in Fig. 6. (a) is relatively long so as to collect accurate information, and therefore enlarge the drift effect.
